# Sugar as a Dressing for Wounds

**Published:** 1884-01

**Authors:** 


### Sugar as a Dressing for Wounds.

Dr. F. Fischer, writing in the *Centralblatt fur Chirurgie*, says that in the Strasburg Hospital, where he is assistant in the surgical department, Professor Lucke has since May used powdered cane-sugar as an antiseptic dressing to wounds. Hitherto, it has been used in combination with naphthalin (equal parts) or with iodoform (1 part to 5 of sugar). In cases of wounds united by suture, the mixture is put up in gauze and applied to the part; where there is loss of skin, the sugar is sprinkled direct over the part. The sugar-dressing is fixed in place by some layers of gauze deprived of fat, over which a layer of gutta-percha is applied, and the whole is secured by a bandage. The sugar-dressing may remain from eight to fourteen days, without the sugar dissolving; the secretion from the wound is equally distributed through the sugar, and it is only when the layer of sugar is too thick (more than about one-fifth of an inch) that lumps are formed. The wounds have a healthy appearance under the sugar, the dressings are not offensive, and bacteria cannot be found in them. Healthy granulations, with no tendency to bleed, are developed; and cicatrization proceeds rapidly. In wounds united by suture, healing by the first intention has always been observed. Dr. Fischer is not able to say whether the sugar is decomposed, or what new products are formed. Writing in the *Allgemeine Medicinische Central-Zeitung* in reference to Dr. Fischer's communication, Dr. Windelschmidt, of Cologne, says that he has used sugar alone as a dressing, with good results. He finds that for small wounds and ulcers powdered cane-sugar is not inferior to iodoform as a dressing, while iodoform is necessary in many cases such as chancres and mammary abscesses. He calls attention to the fact that powdered sugar is a very old popular remedy in cases of fungous granulations, ichorous eczema, and erysipelas of the face. Whether the action of sugar is antiseptic, is not certain; that it is aseptic is proved by its use in confectionary. In spite of his success, Dr. Windelschmidt has for several months desisted from the use of sugar as a dressing; partly, because the patients found out the nature of the powder that was applied and ceased to trust in it; partly, because they treated themselves and so passed from his observation. He thinks, however, that sugar, like iodoform and naphthalin, has its sphere of application; and joins with Dr. Fischer in recommending that it be more extensively tried.—*British Medical Journal.*

---

MEDICAL Inspector, A. C. Gorgas, in his report upon the Naval Academy, at Annapolis, says in reference to the use of tobacco:

"Since my last sanitary report I have had abundant occasion to verify all that has been said against the effects of tobacco upon growing youths. . . I found among the cadets instances of its effects upon muscular co-ordinative power and upon the action of the heart, which more than confirmed my own former observations and the statements of others upon the subject. In the drawing department of this school, it has long been recognized that excessive smoking impairs the control of muscular co-ordination, and the head of that department has been able to detect the smokers by the character of their work in the sketching room." . . . .

" One's Two."—In came a gentleman and sat down, and says to the man-waiter, very nice and gentle: " Have you some nice Providence River Oysters?" " Oh, yes," says the waiter. " Real nice ones, now?" says the gentleman. " Oh, why certainly," says the waiter. " Well, I wish you would open for me a dozen, please." " All right, sir," says the waiter, and he was coming away. " Wait a bit," says the gentleman; " is the butter nice and sweet?" " We have some powerful fresh butter," says the waiter. " Do you have nice fresh milk?" said the gentleman, " Well, its generally so considered," says the waiter. " Well. how are your crackers, nice and fresh!" says the gentleman. " Never had no fault found with our crackers," says the waiter. " Then if you'll take and make me up a nice little stew, John, I'll be much obliged to you," says the gentleman. Then he let him go. When I saw him coming, I says to myself, says I, " How on earth will that man remember all that 'ere?" But he marched right up to the pipe, and just opened his mouth, and says: " One's Two," and that was all.—*Boston Globe.*

